# Remotely Supervised Exercise during the COVID-19 Pandemic versus in-Person-Supervised Exercise in Achieving Long-Term Adherence to a Healthy Lifestyle

**DOI:** 10.3390/ijerph182212198

**Published:** 2021-11-20

**Authors:** Guillermo García Pérez de Sevilla, Olga Barceló Guido, María de la Paz De la Cruz, Ascensión Blanco Fernández, Lidia B. Alejo, María Isabel Ramírez Goercke, Margarita Pérez-Ruiz

**Affiliations:** 1 Department of Physiotherapy, Faculty of Sport Sciences, Universidad Europea de Madrid, 28670 Madrid, Spain; 2 Faculty of Sport Sciences, Universidad Europea de Madrid, 28670 Madrid, Spain; olga.barcelo@universidadeuropea.es (O.B.G.); lidia.brea@universidadeuropea.es (L.B.A.); margarita.perez@universidadeuropea.es (M.P.-R.); 3 Medical Service, Universidad Europea de Madrid, 28670 Madrid, Spain; mariadelapaz.delacruz@universidadeuropea.es; 4 Faculty of Biomedical and Health Sciences, Universidad Europea de Madrid, 28670 Madrid, Spain; ascension.blanco@universidadeuropea.es (A.B.F.); marissa89_8@hotmail.com (M.I.R.G.)

**Keywords:** virtual, exercise, Mediterranean diet, COVID-19, lifestyle, adherence, workplace, quality of life

## Abstract

The World Health Organization’s global action plan on workers’ health establishes that occupational health services should carry out lifestyle interventions within the workplace, to prevent the development of non-communicable diseases. The objective of the study was to compare adherence to a healthy lifestyle six months after completion of a multi-component intervention with remotely supervised physical activity during the COVID-19 pandemic versus a multi-component intervention with in-person supervised physical exercise before the COVID-19 pandemic in university employees with unhealthy habits and predisposed to change. A prospective cohort study following the “Strengthening the Reporting of Observational studies in Epidemiology” (STROBE) guidelines was conducted, with two arms. Each multi-component intervention lasted for 18 weeks, and consisted of education on healthy habits, Mediterranean Diet (MedDiet)-based workshops, and a physical exercise program. Twenty-one middle-aged sedentary university employees with poor adherence to the MedDiet completed the study. Six months after completion of the intervention, both groups increased physical activity levels, adherence to the MedDiet, eating habits, health-promoting lifestyle, health responsibility, and health-related quality of life. There were no differences between groups in any of the variables analyzed. Therefore, remotely supervised physical exercise could be adequate to achieve long-term adherence to a healthy lifestyle in the same way as conventional face-to-face intervention, at least in a population willing to change.

## 1. Introduction

Recently, it has been highlighted that a healthy lifestyle is an effective strategy to improve health and reduce the incidence of non-communicable diseases (NCDs), like type 2 diabetes or cardiovascular diseases [1]. Among the behaviors that define a healthy lifestyle, the most important factors are physical activity (PA) and diet [2]. The protective effect of physical exercise against NCDs depends on the dose [3], and in this sense, the World Health Organization (WHO) proposes general recommendations for the promotion of PA worldwide in 2020 to prevent NCDs [4]. Concerning eating habits, the Mediterranean diet is a healthy pattern characterized by a low intake of saturated fat and salt due to the low consumption of red and ultra-processed meats; high consumption of monounsaturated fatty acids (MUFA) due to olive oil and nuts; an adequate balance of polyunsaturated fatty acids (PUFA) thanks to nuts, fatty fish, and green leafy vegetables; and high consumption of antioxidants and fiber, due to the high intake of fruits and vegetables [5]. This eating pattern has traditionally been associated with a protective effect against cardiometabolic diseases [6,7]. 

Most exercise and diet obesity interventions successfully achieve their objectives but do not maintain these changes in the long term, and after six months, generally, they lose effectiveness [8]. Six months is the minimum time necessary to achieve adherence to new lifestyle habits [9]. The same occurs with virtual interventions that assess long-term adherence to PA levels [10] or sedentary behavior [11]. Therefore, poor adherence to lifestyle changes, which sometimes is due to lack of motivation or misunderstanding of the risks of unhealthy habits, is a frequent obstacle in exercise and diet interventions [12]. 

To solve the frequent poor adherence to lifestyle interventions, a meta-analysis performed in 2018 highlights the importance of having psychological assistance [13]. In this sense, the transtheoretical model of behavior change (TTM) proposes that there are six stages of change in which the patient can be, which mark the preparation of the participants to perform lifestyle changes [9]. The optimal stage for incorporating healthy lifestyle habits is the contemplation stage, in which participants are in a state of predisposition to change [14]. According to Howlett et al., the TTM is an effective strategy in physical exercise interventions to achieve significant results in changing exercise habits and maintaining them after six months, through experiential and behavioral processes of change, although the size effect is low [15]. Giving information to the patients about their health status and the impact that lifestyle has on the possible development of NCDs is another effective strategy to maintain lifestyle changes [16]. 

The COVID-19 pandemic has implied a reorganization of our society and our lifestyle, with mobility restrictions, so virtual interventions are appropriate in this context. Furthermore, sedentary behavior levels have increased during the COVID-19 pandemic [17,18,19,20,21]. Interventions based on physical exercise through digital platforms have been shown to be appropriate to reach the PA recommendations of the WHO in some populations [22]. However, according to a recent systematic review, there is modest evidence regarding the effectiveness of virtual interventions in the workplace on health-related outcomes [23].

Another advantage of virtual interventions is that they have lower costs, greater time flexibility, and higher time savings by not having to travel, compared to face-to-face interventions, favoring adherence [24]. However, to date, there are no studies reported that compare in-person versus remotely supervised physical exercise and diet interventions, so it is not known whether remotely supervised interventions are as effective as face-to-face interventions at achieving adherence to healthy lifestyle habits.

Healthy lifestyles help to prevent the development of NCDs, and the work environment covers an age group with silent risk factors such as high levels of sedentary behavior [25]. In this sense, the WHO global action plan on workers’ health establishes that occupational health services should carry out lifestyle interventions within the workplace [26]. In addition, health promotion interventions within the companies improve productivity and reduce absenteeism and medical expenses [21,27]. Also, a healthier lifestyle is associated with less perceived stress, increased mental health, and greater productivity in the workplace [28]. As well, in observational studies positive relationships have been found between high adherence to the Mediterranean diet and a higher Health-related quality of life (HrQoL) [29] and between high PA levels and a higher HrQoL [30,31,32,33,34]. High quality randomized controlled trials have reported significant improvements of HrQoL after workplace exercise interventions, although without a long-term follow-up post-intervention [35,36].

Very few randomized clinical trials with Mediterranean diet interventions within the workplace managed to increase adherence to this diet pattern, although without long-term follow-up [37,38]. Regarding PA, only one study managed to increase and maintain PA levels six months after in-person physical exercise intervention [39]. Another recent randomized clinical trial with a remotely supervised physical exercise and diet intervention reported a long-term improvement in physical exercise and eating habits, although without significantly increasing weekly metabolic equivalent tasks (METS)-min or adherence to the Mediterranean diet [40]. 

The objective of this study was to analyze whether remotely supervised physical exercise is as effective in achieving adherence to a healthy lifestyle and enhancing HrQoL as in-person supervised physical exercise in employees predisposed to change. This study hypothesized that in employees who are predisposed to change, a remotely supervised physical exercise and diet intervention achieves similar adherence to a healthy lifestyle and similar improvement in HrQoL as a physical exercise and diet intervention with in-person supervision.

## 2. Materials and Methods

### 2.1. Study Design

A prospective cohort study was conducted, with two lifestyle multi-component interventions (in-person supervised exercise cohort and remotely supervised exercise cohort), following the statement “Strengthening the Reporting of Observational studies in Epidemiology” (STROBE) [41].

The study protocol adheres to the “Ethics Guidelines of the Declaration of Helsinki”, the last modification produced in 2011. It has the approval of the regional ethics committee of the Community of Madrid (CEIm) (record nº 05/20, EC 42/19). All participants signed informed consent before participating in the study.

### 2.2. Participants

The participants of both cohorts were university employees that were selected by convenience sampling from the occupational health service. At two different times, December 2018 and December 2019, informative meetings of the intervention were held. To see if they met the inclusion criteria, employees interested in participating in the intervention filled out the Mediterranean Diet Adherence Score (MEDAS), the University of Rhode Island Change Assessment Scale (URICA), and the Global Physical Activity Questionnaire (GPAQ).

The inclusion criteria were the following: (1) University employees, adults; (2) Not complying with 2010 the WHO PA recommendations [42]; (3) Having a score inferior or equal to 9 in the MEDAS questionnaire, which means poor adherence to the Mediterranean diet [43]; (4) being in the contemplation stage, which means a predisposition to change, according to the URICA questionnaire [44].

The exclusion criteria were having chronic diseases or musculoskeletal injuries that contraindicate physical exercise. 

### 2.3. Lifestyle Intervention 

The first cohort, the in-person supervised exercise group (ISEG) started in January 2019 a multi-component intervention. Before that, the participants received a detailed report on their lifestyle habits such as PA, sedentary behavior, or diet, and the possible long-term consequences on their health. In the first place, an educational program on healthy habits was carried out in which the participants viewed 12 weekly videos on different topics: (1) Motivation for change; (2) Nutrients, fiber and water; (3) Frequency of eating; (4) Breakfast and snacking; (5) From the food market to your dining table; (6) Circadian rhythms; (7) Physical activity recommendations; (8) False food and physical exercise myths; (9) Body composition healthy values; (10) Chronic diseases; (11) Nutritional strategies; (12) Physical exercise strategies. Three weeks after starting this first component, the diet program started in parallel, consisting of nine face-to-face weekly healthy eating workshops of 90 mins duration. In these workshops, the participants practiced diet planning following the Mediterranean diet pattern, addressed barriers to change, and reinforced the nutritional concepts of the educational program. Once the nutrition component was finished, an in-person-supervised physical exercise program was carried out, lasting for lasted six weeks, with 18 sessions of 60 mins each, with a frequency of three weekly sessions, combining strength and resistance exercises, and following 2010 WHO recommendations.

The typical training session consisted of a 10-min warm-up of mobility exercises; a 40-min main part consisting of a strength circuit training with 2–3 sets of 12–15 repetitions of 7–8 exercises involving major muscle groups, at an intensity of 7 to 8 RPE, with a rest of 30 s between exercises and 1 min between sets, and then aerobic exercise (treadmill walking or stationary cycling) at an intensity of 7 to 8 on the revised category-ratio Borg scale of perceived exertion (RPE); and a 10-min cool down consisting of flexibility exercises.

The second cohort, the remotely supervised exercise group (RSEG), started the same program in January 2020. The difference was that at the end of the diet component, the Spanish government ordered a strict stay-at-home lockdown to stop the expansion of the COVID-19 outbreak, so the physical exercise program was remotely supervised in real-time, as the participants were at home. In this exercise program, with the same characteristics as that of the ISEG, the participants performed self-loading strength exercises and performed aerobic exercise jogging at home since they did not have specific training material or large spaces [40]. 

**Figure 1:**
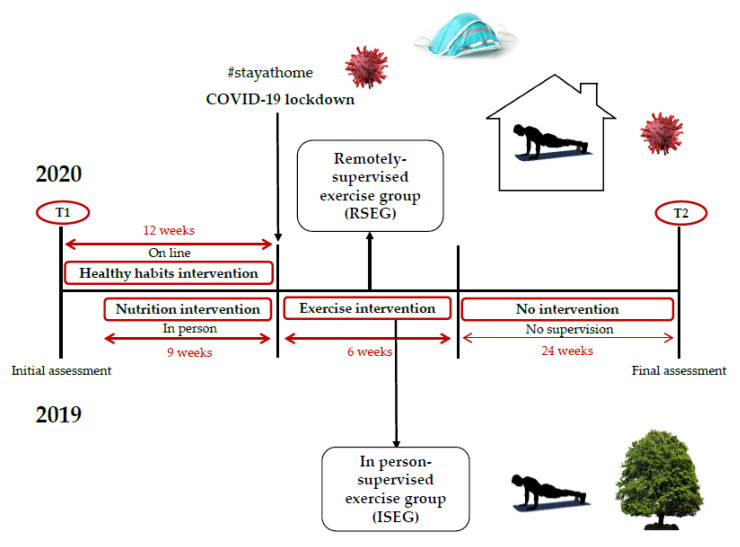
Organization of The Two Lifestyle Interventions.

For each participant, the diet workshops were in-person supervised by two nutritionists for both cohorts, while the physical exercise programs were supervised by two PA professionals, in-person (ISEG) and remotely (RSEG) (Figure 1).

### 2.4. Place of the Intervention and Times of Assessments

The diet workshops were carried out at the university campus facilities during the employees’ workday for the ISEG and RSEG. The ISEG performed the PA program at the university campus facilities, while the RSEG performed it at home, with remote supervision, due to the COVID-19 lockdown. Both cohorts were evaluated before the multi-component intervention (T1) and six months after completion of it (T2).

### 2.5. Variables

#### 2.5.1. Lifestyle

To analyze lifestyle, the Health-Promoting Lifestyle Profile II (HPLP-II) questionnaire was filled out. It consists of 52 items that are answered as N (never, 1 point), S (sometimes, 2 points), O (Often, 3 points), and R (Routinely, 4 points). This questionnaire consists of six subscales: Health responsibility (9 items), Physical activity (8 items), Nutrition (9 items), Spiritual growth (9 items), Interpersonal relations (9 items), and Stress management (8 items). The score for each scale is calculated using the means of the items [45,46]. Concerning the health-promoting lifestyle total score, the minimum is 52 and the maximum 208. It is considered a low score and therefore an inadequate lifestyle 52–90, moderate score and an improvable lifestyle 91–129, good lifestyle 130–168, and excellent lifestyle 169–208 [47]. 

To analyze another of the specific components of lifestyle, the MEDAS questionnaire was filled out to assess adherence to the Mediterranean diet. This questionnaire consists of 14 items, of which each adds 0 or 1 point. The items of this questionnaire positively evaluate the consumption of vegetables, fruit, olive oil, fish, nuts, and negatively the consumption of red meat, sugary drinks, commercial pastries, or butter. It is considered high adherence ≥10 points, which is a strong protector against cardiovascular diseases, medium adherence 8 to 9 points, and poor adherence ≤7 points [43]. 

PA level and sedentary behavior were analyzed using the GPAQ questionnaire [48,49]. This questionnaire analyzes the daily sitting hours, as well as the level of PA in the categories of work, journey, and leisure time, allowing estimating energy expenditure in weekly METs-min and classifying the subjects into three categories: Category 1: Low/inactive, which means not meeting the criteria of categories 2 or 3. Category 2: Moderate, which means accumulating 600 METs-min per week of moderate-intensity PA spread over 5–7 days per week. Category 3: High, which means accumulating 1500 METs-mins per week of vigorous-intensity PA spread over 3–7 days per week; or 3000 MET-mins per week of moderate to vigorous-intensity PA spread over the 7 days of the week [4]. 

#### 2.5.2. Health-Related Quality of Life

To analyze HrQoL, the Short Form 36 Health Survey Questionnaire v2 (SF36) was used, which consists of 36 items scored from 0 (worst perception of HrQoL) to 100 (best perception of HrQoL) in 8 health concepts: Physical Function, Role Physical, Bodily Pain, General Health, Vitality, Social Functioning, Role Emotional, and Mental health [50]. 

These eight health concepts are regrouped into the Physical Component Summary and the Mental Component Summary. A 4-point increase in any of these two components is considered clinically relevant in healthy adults [51].

#### 2.5.3. Anthropometric Variables

Height (cm, Ano Sayol SL height rod, Barcelona, Spain), and weight (kg, Asimed T2 scale, Barcelona, Spain) were measured. Then, the body mass index (BMI, in kg/m^2^) was calculated.

### 2.6. Bias

Possible biases in this study could arise from the absence of randomization and a control group so that other external factors beyond the interventions could influence the results. To reduce this risk of bias, the primary analysis of this study was comparing the results obtained by both interventions in lifestyle and HrQoL at T2 to see whether there were significant differences between both cohorts.

### 2.7. Sample Size

The sample size was calculated using data from a pilot study, where the primary variable was the effect on lifestyle according to the HPLP-II questionnaire, with an alpha error of 0.05 and a beta error of 0.2. Using the G-Power v.3.1 software (Erdfelder et al., Kiel, Germany), the resulting sample needed to achieve the objective of the study was 22 participants, so 24 participants were sampled to compensate for a probable 10% dropout.

### 2.8. Statistical Analysis

All the results were analyzed by protocol and intention-to-treat analysis (ITT). The distribution and normality of the data were analyzed with the Shappiro–Wilk and Levene tests and with P-P and Q-Q plots. Data are expressed as mean ± SD. The independent T test and the Mann–Whitney U-test were used to compare the differences between both groups (RSEG and ISEG) before the multi-component intervention, with the aim of evaluating the homogeneity of the groups. Then, a paired *t*-test was performed for both ISEG and RSEG, to determine the time difference. Finally, to compare the difference between the results of the two interventions, the independent *t*-test and the Mann–Whitney U-test were used again. The level of statistical significance was set at *p* < 0.05. Eta partial squared (*η*^2^*p*) was used as a measure of effect size [52], considering 0.01 a low effect size, 0.06 a moderate effect size, and 0.14 a large effect size [53]. All statistical analysis was performed with SPSS 27.0 (IBM, Armonk, NY, USA).

## 3. Results

### 3.1. Recruitment

**Figure 2:**
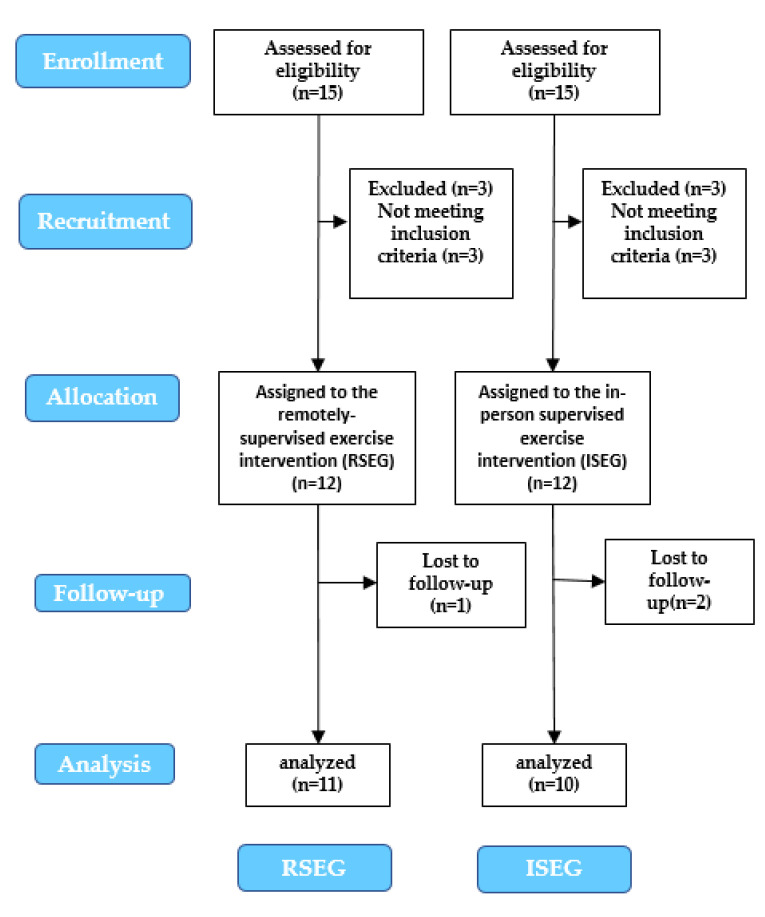
Flow Diagram of The Two Multi-Component Interventions.

Of 15 participants initially recruited in January 2019, three (20%) did not met the inclusion criteria, so n = 12 subjects were assigned to the ISEG. Also, of 15 participants initially recruited in January 2020, three (20%) did not met the inclusion criteria, so n = 12 subjects were assigned to the RSEG. There was one dropout in the RSEG, and two dropouts in the ISEG, so the final analysis was performed on 11 RSEG and 10 ISEG subjects, as is shown in the flow diagram (Figure 2). This study ended six months after completion of the intervention to assess long-term adherence.

### 3.2. Description of the Sample

In the RSEG, 42% of the subjects were men and 58% women, while in the ISEG, 25% were men and 75% were women. The mean age of the RSEG was 42.78 ± 6.88 years, the bodyweight was 74.98 ± 15.68 kg, and the BMI was 25.82 ± 3.70 kg/m^2^; while the mean age of the ISEG was 43.35 ± 7.59 years, the weight was 77.89 ± 13.88 kg, and the BMI was 27.63 ± 4.64 kg/m^2^. There were no significant differences in these variables between the two groups.

### 3.3. Lifestyle

In the T1-T2 analysis, the RSEG participants significantly improved their lifestyle, both in the total score of the HPLP II questionnaire (121.27 ± 12.54, vs. 141.73 ± 17.43; *p* < 0.001; *η*^2^*p* = 0.68), and in the categories of Health Responsibility (1.93 ± 0.31, vs. 2.37 ± 0.47; *p* < 0.01, *η*^2^*p* = 0.67), Physical Activity (1.73 ± 0.51, vs. 2.48 ± 0.54; *p* < 0.001, *η*^2^*p* = 0.72), Nutrition (2.46 ± 0.31, vs. 2.96 ± 0.57; *p* < 0.01, *η*^2^*p* = 0.65), Spiritual Growth (2.76 ± 0.31, vs. 3.03 ± 0.32; *p* = 0.01, *η*^2^*p* = 0.48), and Stress Management (2.07 ± 0.33, vs. 2.38 ± 0.43; *p* = 0.02, *η*^2^*p* = 0.42), with a large effect size for these six variables. Also, the RSEG participants significantly improved their Adherence to the Mediterranean diet (7.00 ± 1.41, vs. 9.82 ± 1.60; *p* <0.001, *η*^2^*p* = 0.73), with a large effect size, progressing from low adherence (MEDAS score ≤7) to medium adherence (MEDAS score 8–9). The RSEG participants also increased significantly their PA levels (327.27 ± 258.96, vs. 1327.21 ± 1045.15 METS-min/week; *p =* 0.01, *η*^2^*p* = 0.47), progressing from low to moderate levels [4], with a large effect size. Finally, the daily sitting time decreased 2.5 h in the RSEG, although without significance (*p* = 0.06) (Table 1).

In the T1-T2 analysis, the ISEG participants significantly improved their lifestyle, both in the total score of the HPLP II questionnaire (121.30 ± 11.10, vs. 137.20 ± 9.48; *p* < 0.01; *η*^2^*p* = 0.57), and in the categories of Health Responsibility (2.06 ± 0.38, vs. 2.36 ± 0.33; *p* = 0.02, *η*^2^*p* = 0.49), Physical Activity (1.43 ± 0.32, vs. 2.09 ± 0.51; *p* < 0.01, *η*^2^*p* = 0.57), and Nutrition (2.35 ± 0.40, vs. 2.84 ± 0.57; *p* = 0.02, *η*^2^*p* = 0.47), with a large effect size for these four variables. Also, the ISEG participants significantly improved their Adherence to the Mediterranean diet (7.00 ± 1.56, vs. 9.10 ± 2.08; *p =* 0.01, *η*^2^*p* = 0.53), with a large effect size, progressing from low adherence (MEDAS score ≤7) to medium adherence (MEDAS score 8–9). The ISEG participants also increased significantly their PA levels (28.00 ± 59.78, vs. 1160.00 ± 913.31 METS-min/week; *p <* 0.01, *η*^2^*p* = 0.43), progressing from low to moderate levels [4], with a large effect size. Finally, the daily sitting time significantly decreased (612.00 ± 132.06, vs. 537.00 ± 144.53 min; *p* = 0.03, *η*^2^*p* = 0.44) (Table 1).

When comparing the results in the T1-T2 analysis between the RSEG and the ISEG, there were not significant differences in any of the lifestyle variables analyzed (Table 2).

### 3.4. Health-Related Quality of Life

In the T1-T2 analysis, the RSEG participants significantly improved their HrQoL in the cathegories of Vitality (66.36 ± 17.62, vs. 82.27 ± 9.05; *p* < 0.01, *η*^2^*p* = 0.64), General Health (68.45 ± 10.88, vs. 83.82 ± 9.82; *p* < 0.01, *η*^2^*p* = 0.63), and the Physical Component Summary (49.06 ± 5.04, vs. 54.51 ± 4.02; *p* = 0.01, *η*^2^*p* = 0.49), with a large effect size for these three variables. Regarding the Physical Component Summary, the improvement was clinically significant (>4 points) [51] (Table 1).

In the T1-T2 analysis, the ISEG participants significantly improved their HrQoL in the cathegory of General Health (73.00 ± 9.07, vs. 84.50 ± 7.55; *p* < 0.01, *η*^2^*p* = 0.65), with a large effect size. In the case of the Mental Component Summary, there was a clinically significant improvement (>4 points) [51], although it was not statistically significant (*p* = 0.32) (Table 1).

When comparing the results in the T1-T2 analysis between the RSEG and the ISEG, there were not significant differences in any of the HrQoL variables analyzed (Table 2).

### 3.5. Anthropometric Variables

Bodyweight did not change in the T1-T2 analysis in the RSEG (*p* = 0.24), but significantly decreased in the ISEG (77.68 ± 13.26, vs. 73.28 ± 11.17; *p* < 0.01, *η*^2^*p* = 0.63), with a large effect size (Table 1). There were not significant differences between the RSEG and ISEG (Table 2).

### 3.6. Compliance with the Intervention

Compliance was high in both groups. In the RSEG, the mean attendance was 92% for the physical exercise sessions, and 84% for the nutritional workshops. Concerning the ISEG, the mean attendance was 95% for the physical exercise sessions, and 88% for the nutritional workshops. There were no adverse effects caused by the intervention.

## 4. Discussion

The objective of the present study was to compare adherence to a healthy lifestyle six months after completion of a multi-component intervention with remotely supervised physical exercise in the context of the COVID-19 pandemic versus six months after completion of a multi-component intervention with in-person supervised physical exercise intervention before the COVID-19 pandemic in university employees predisposed to change. Six months after completion of the intervention, both groups obtained increases over time in PA levels, adherence to the Mediterranean diet, diet quality, health-promoting lifestyle, health responsibility, and HrQoL. There were no differences between both groups in any of the variables analyzed. Compliance was high, around 90%, both in the physical exercise sessions and in the nutritional workshops, in the RSEG and ISEG participants.

Both groups improved PA levels six months after completion of the intervention, qualitatively (HPLP-II questionnaire) and quantitatively (GPAQ questionnaire), with a large effect size, progressing from low PA levels to medium PA levels, with no significant differences between groups. These data contrast with a systematic review conducted in 2016 by Schoeppe et al. [10], in which they reported that virtual interventions were not effective in increasing PA levels. Possibly, the fact that all the participants were predisposed to change and were always supervised by a professional could have influenced the results of the present study [13,14,15]. On the other hand, both groups increased PA levels in the same way, even though during the COVID-19 pandemic in which the RSEG participants were, PA levels decreased among the general population [19,20,54]. 

Regarding sedentary behavior, the ISEG decreased the daily sitting hours, with a large effect size, while the RSEG did not. These data are similar to a systematic review carried out in 2017 by Stephenson et al., in which virtual interventions failed to reduce sedentary behavior [11]. However, there were no significant differences between groups, even though during the COVID-19 pandemic in which the RSEG participants were, sedentary behavior increased among the general population [17,18,19,20,21].

Both groups increased adherence to the Mediterranean diet, with large effect size, and acquired healthy habits such as following a diet low in saturated fat, limiting the consumption of sugars and sweets, eating 2–4 servings of fruit and 3–5 servings of vegetables a day, or limiting the consumption of salt, according to the HPLP-II questionnaire. Participants probably spent less time eating at restaurants and more time cooking at home. Both diet components were similar as they consisted of in-person workshops. The difference between groups was the context of the COVID-19 pandemic in which the RSEG participants were. The ISEG and the RSEG showed similar improvements, without statistically significant differences, so the context generated by the COVID-19 pandemic was not decisive in these variables to implement this change in habits, even though during this time the prevalence of snacking between meals and the consumption of alcoholic beverages and carbonated beverages have increased [19,54]. Other studies, however, have described that during the COVID-19 pandemic, adherence to the Mediterranean diet increased among the general population [55]. Also, along with our study, other interventions carried out in the workplace have increased adherence to the Mediterranean diet [37,38], increasing the consumption of MUFA, PUFA, and reducing the consumption of cholesterol and saturated fats, although without performing a long-term post-intervention follow-up.

As well as the participants of the RSEG and the ISEG improved their PA levels and their eating habits, their HrQoL also increased. In this regard, some observational studies have found positive correlations between HrQoL and PA levels [31] and adherence to the Mediterranean diet [29], while others do not [56]. The RSEG participants improved the specific categories of HrQoL of vitality, general health, and physical component summary over time with a large effect size for these three variables, while the ISEG improved the general health with a large effect size. No significant differences were found between both interventions, so both groups showed a similar improvement in HrQoL, even though during the COVID-19 pandemic the university community presented very low levels of HrQoL [56]. 

The participants in this study achieved long-term adherence to a healthy lifestyle, which could be partly because they were predisposed to change [14], and had psychological support since nutritional barriers and motivation to change were addressed during the workshops [13,15]. Also, before starting the intervention the participants became aware of their unhealthy lifestyle habits and their possible repercussions on the development of NCDs through a detailed report that was given to them [16].

The clinical relevance of the study is that it involves multi-component interventions that promote a healthy lifestyle within the workplace, from the occupational health service, as established by the WHO [26], in employees with risk factors for the development of NCDs, such as sedentary behavior and overweight. Furthermore, long-term improvement in lifestyle is associated with lower stress levels, better mental health, and higher work productivity [28] Some limitations of this study are that the variables analyzed were self-reported validated surveys and that the population was in a state of predisposition to change, so these results cannot be extrapolated to a population that is not willing to change. The effects achieved in each cohort cannot be attributed to the interventions since the present study was not a randomized controlled trial. On the other hand, the confinement due to the COVID-19 pandemic made it necessary to adapt the physical exercise intervention of the second cohort to virtual supervision.

## 5. Conclusions

In this study carried out on overweight middle-aged university employees within the workplace, both groups showed similar long-term improvements in their HrQoL and lifestyle, mainly in PA levels and eating habits. The context of the COVID-19 on which was the RSEG did not seem to have influenced the results. Therefore, remotely supervised physical exercise could be adequate to achieve these objectives in the same way as a conventional face-to-face intervention, at least in a population willing to change.

## Figures and Tables

**Table 1:** Analysis of Lifestyle and Health-Related Quality of Life in The Two Interventions.

Variables	Group	T1	T2	*p*-Value Time	*η*^2^*p* Time
**HPLP II**					
Health-Promoting Lifestyle Profile total score	RSEG ISEG	121.27 ± 12.54 121.30 ± 11.10	141.73 ± 17.43 137.20 ± 9.48	<0.001 * <0.01 *	0.68 0.57
Health Responsibility	RSEG ISEG	1.93 ± 0.31 2.06 ± 0.38	2.37 ± 0.47 2.36 ± 0.33	<0.01 * 0.02 *	0.67 0.49
Physical Activity	RSEG ISEG	1.73 ± 0.51 1.43 ± 0.32	2.48 ± 0.54 2.09 ± 0.51	<0.001 * <0.01 *	0.72 0.57
Nutrition	RSEG ISEG	2.46 ± 0.31 2.35 ± 0.40	2.96 ± 0.57 2.84 ± 0.57	<0.01 * 0.02 *	0.65 0.47
Spiritual growth	RSEG ISEG	2.76 ± 0.31 2.80 ± 0.38	3.03 ± 0.32 3.12 ± 0.43	0.01 * 0.18	0.48 0.19
Interpersonal Relations	RSEG ISEG	3.06 ± 0.42 3.01 ± 0.43	3.07 ± 0.43 3.24 ± 0.34	0.65 0.14	0.02 0.23
Stress management	RSEG ISEG	2.07 ± 0.33 1.94 ± 0.51	2.38 ± 0.43 2.17 ± 0.44	0.02 * 0.30	0.42 0.12
**MEDAS**					
Adherence to the Mediterranean diet	RSEG ISEG	7.00 ± 1.41 7.00 ± 1.56	9.82 ± 1.60 9.10 ± 2.08	<0.001 * 0.01 *	0.73 0.53
**GPAQ**					
Physical activity levels (METS-min per week)	RSEG ISEG	327.27 ± 258.96 28.00 ± 59.78	1327.27 ± 1046.15 1160.00 ± 913.31	0.01 * <0.01 *	0.47 0.63
Daily sitting time (min)	RSEG ISEG	463.64 ± 180.18 612.00 ± 132.06	312.73 ± 150.80 537.00 ± 144.53	0.06 0.03 *	0.31 0.44
**SF-36**					
Physical Component Summary	RSEG ISEG	49.06 ± 5.04 51.32 ± 5.11	54.51 ± 4.02 53.35 ± 4.15	0.01 * 0.31	0.49 0.12
Mental Component Summary	RSEG ISEG	51.43 ± 8.24 48.03 ± 15.04	53.07 ± 5.99 53.16 ± 8.97	0.45 0.32	0.05 0.11
**ANTHROPOMETRY**					
Body weight (kg)	RSEG ISEG	79.32 ± 13.19 77.68 ± 13.26	76.89 ± 14.86 73.28 ± 11.17	0.24 0.01 *	0.15 0.63

T1, initial assessment; T2, final assessment; RSEG, remotely supervised exercise group; ISEG, in-person supervised exercise group; *η*^2^*p*: effect size in time. Differences between time interactions were evaluated using a paired *t*-test. Significance was set at <0.05 *.

**Table 2:** Between-Group Comparisons in Total Change in Lifestyle and Health-Related Quality Variables.

Variables	RSEG	ISEG	*p*-Value
**HPLP II**			
Health-Promoting Lifestyle Profile total score	+20.45 ± 14.60	+15.90 ± 14.40	0.43
Health Responsibility	+0.49 ± 0.36	+0.26 ± 0.26	0.12
Physical Activity	+0.73 ± 0.48	+0.69 ± 0.63	0.87
Nutrition	+0.54 ± 0.41	+0.43 ± 0.49	0.61
Spiritual growth	+0.29 ± 0.32	+0.24 ± 0.53	0.80
Interpersonal Relations	+0.05 ± 0.36	+1.18 ± 0.34	0.42
Stress management	+0.30 ± 0.37	+0.16 ± 0.47	0.48
**MEDAS**			
Adherence to the Mediterranean diet	+2.82 ± 1.78	+2.10 ± 2.08	0.40
**GPAQ**			
Physical activity levels (METS-min per week)	+1000.00 ± 1110.60	+1132.00 ± 922.81	0.77
Daily sitting time (min)	−150.90 ± 238.05	−75.00 ± 88.60	0.08
**SF-36**			
Physical Component Summary	+5.45 ± 5.86	+2.03 ± 5.94	0.11
Mental Component Summary	+1.63 ± 7.90	+5.13 ± 15.41	0.52
**ANTHROPOMETRY**			
Body weight (kg)	−2.43 ± 6.09	−4.40 ± 3.53	0.08

RSEG, remotely supervised exercise group; ISEG, in-person supervised exercise group. Differences between groups were evaluated using an independent T-test and the Mann–Whitney U-test. Significance was set at <0.05.

## Data Availability

Data available upon request due to ethical and privacy restrictions.
